# *TNRC9* rs12443621 and *FGFR2* rs2981582 polymorphisms and breast cancer risk

**DOI:** 10.1186/s12957-016-0795-7

**Published:** 2016-02-24

**Authors:** Ying Chen, Chunying Shi, Qiyong Guo

**Affiliations:** Department of Radiology, Shengjing Hospital Affiliated to China Medical University, No. 36, Sanhao ST, Heping District, Shenyang, Liaoning Province 110004 China; Department of Radiology, First Affiliated Hospital of Harbin Medical University, No. 23 Post ST, Nangang District, Harbin, Heilongjiang Province 150081 China

**Keywords:** *FGFR2*, *TNRC9*, *LSP1*, SNP, Breast cancer mammographic density

## Abstract

**Background:**

This study is to investigate the association of fibroblast growth factor receptor 2 (*FGFR2*) rs2981582, trinucleotide-repeat-containing 9 (*TNRC9*) rs3803662, rs12443621, and leukocyte-specific protein 1 (*LSP1*) rs3817198 polymorphisms with breast cancer and mammographic density in Han Chinese population.

**Methods:**

TaqMan Single Nucleotide Polymorphism (SNP) Genotyping Assays and unconditional logistic regression analysis were used to examine these SNPs in 105 breast cancer cases and 382 controls.

**Results:**

The genotype frequencies of rs12443621 and rs2981582 were significantly different between controls and cases (*P* = 0.017 and 0.006, respectively). Subjects carrying G allele of rs12443621 had increased breast cancer risk (AG vs AA: OR = 2.017, 95 % CI = 0.910–4.471; GG vs AA: OR = 2.684, 95 % CI = 1.318–5.463). Subjects carrying an allele of rs2981582 had reduced breast cancer risk (GA vs GG: OR = 0.444, 95 % CI = 0.262–0.752; AA vs GG: OR = 0.579, 95 % CI = 0.342–0.983). rs3803662 and rs3817198 SNPs did not significantly differ between cases and controls (*P* = 0.408 and 0.116, respectively). Interestingly, the AA genotype of rs2981582 was also associated with reduced mammographic densities (*P* = 0.0092, 95 % CI = 0.334–0.926).

**Conclusions:**

Our findings indicate that the GG genotype of rs12443621 is associated with increased breast cancer risk whereas the GA and AA genotypes of rs2981582 are reduced risk in Han Chinese population.

## Background

Breast cancer is one of the most common malignancies and a leading cause of cancer death in women worldwide, with over one million new cases each year [1]. In China, breast cancer has increased rapidly in recent years and become the most common cancer for women in major cities [2]. In Heilongjiang Province, which is located in Northeast China, there are nearly 6000 new cases of breast cancer every year accounting for 15 % of female malignancies in the area [3]. Therefore, it is necessary to identify risk factors for breast cancer in this Chinese population.

The development and progression of breast cancer is a multi-step complicated process that involves both genetic and epigenetic factors. Although significant advancement has been made in understanding the genetic susceptibility to breast cancer, known susceptibility genes account for less than a quarter of familial breast cancer risk. To further identify novel susceptibility alleles associated with breast cancer, genome-wide association studies (GWAS) have been performed. One of these studies was published on Nature [4], which identifies trinucleotide-repeat-containing 9 (*TNRC9*), fibroblast growth factor receptor 2 (*FGFR2*), mitogen-activated protein kinase 1 (*MAP3K1*), and leukocyte-specific protein 1 (*LSP1*) as four novel loci with consistent evidence of association with breast cancer.

Since then, a number of case-control or cohort studies have been carried out to further examine the association of the single nucleotide polymorphisms (SNPs) with breast cancer, but the results are not completely consistent. For example, a cohort study in American population found that the AG/GG genotypes of *TNRC9* rs12443621 and the CT/TT genotype of *FGFR2* rs2981582 had a decreased risk for death in breast cancer patients [5]. In contrast, another cohort study in Swedish population found that the AG/GG genotypes of *TNRC9* rs12443621 and the AA genotypes of *FGFR2* rs2981582 both had increased breast cancer risk [6]. A case-control study in Tunisian population reported that subjects with AA genotype of *FGFR2* rs2981582 had increased risk of breast cancer [7], but *TNRC9* rs12443621 was not significantly associated with breast cancer risk. Strong ethnic differences have been observed in breast cancer risk. Racial/ethnic differences and environmental factors that vary among populations may affect the associations between SNPs and risk of breast cancer by modulating complex interactions between various genes. Therefore, more studies are needed to clarify the relationship between these SNPs and breast cancer risk.

Mammographic density has been shown to be strongly linked to breast cancer risk [8]. Women with high mammographic density have increased risk for breast cancer as compared to women with lower mammographic density. Because both breast cancer and mammographic density are influenced by genetic factors, they may share some genetic determinants. A recent study showed that women with at least one G allele of rs12443621 in *TRC9* had higher mammographic density than women with two alleles [9]. They also found that women with at least one T allele of rs2981582 in *FGFR2* had non-significantly decreased mammographic density than women with two C alleles.

To have a better understanding of the association of *FGFR2* rs2981582, *TNRC9* rs3803662, rs12443621, and *LSP1* rs3817198 polymorphisms with breast cancer, we investigated these SNPs in Han Chinese women in Heilongjiang Province and analyzed their association with breast cancer risk and mammographic density.

## Methods

### Study population

All samples were collected at the First Affiliated Hospital of Harbin Medical University in 2010 between June and November. A total of 487 participants were recruited in this study. The case group was comprised of 105 female patients with histopathologically diagnosed breast cancer. Patients with a history of cancer, tumor chemotherapy, or radiotherapy were excluded from this study. The control group was comprised of 382 age-matched healthy women. Ninety of the breast cancer cases and 229 of controls had mammographic X-ray. All subjects gave written consent for participation in this research. This study was approved by the Ethical Committee of the First Hospital Affiliated to Haerbing Medical University.

### DNA extraction and TaqMan SNP Genotyping Assays

EDTA-anti-coagulated venous blood samples were preserved at −70 °C. Genomic DNA was isolated from whole blood using the Wizard® kit (Promega, Madison, WI, USA) according to the manufacturer’s protocol. Blinded genotyping of *FGFR2* rs2981582, *TNRC9* rs3803662, rs12443621, and *LSP1* rs3817198 was carried out using TaqMan SNP Genotyping Assays (Applied Biosystems, Foster City, CA, USA) with Mini Option2 Real Time PCR System (BioRad, Hercules, California, USA). Assays were performed with TaqMan Universal Master Mix, TaqMan probe, and 50 ng of DNA per reaction. PCR conditions were provided by the manufacturer: 5 min initial denaturation at 94 °C followed by 45 cycles of 94 °C denaturation for 15 s and 60 °C annealing/extension for 1 min.

### Reproductive factors

Information on age, age at menarche, age at menopause, breast-feeding, parity, and miscarriages/abortions was assessed from the questionnaire. The number of miscarriages/abortions was categorized into three groups: 0, 1–2, and 3 or more.

### Mammographic density

Mediolateral oblique (MLO) and craniocaudal (CC) view digital mammograms were evaluated by three radiologists specialized in mammographic diagnosis. Mammographic density was described by using the Breast Imaging Reporting and Data System (BI-RADS, American College of Radiology) four-category terminology [10]: D1, less than 25 % glandular (category 1), D2, 25–50 % glandular (category 2), D3, 51–75 % glandular (category 3), and D4 greater than 75 % glandular (category 4). The evaluation was blinded to ensure accuracy. For a few films on which there was disagreement in reporting, final reporting was made after the discussion among the radiologists.

### Statistical analysis

Quantitative data were expressed as mean ± SD and analyzed using ANOVA or rank sum test. Categorical data were analyzed using chi-square test. The association between the genotypes and breast cancer risk or mammographic density was evaluated by unconditional logistic regression analysis and expressed as odds ratio (OR) and their 95 % confidence intervals (CI) using SPSS 18.0 software (SPSS Inc., Chicago, IL, USA). Throughout the analysis, a two-sided *P* value less than 0.05 was considered to be statistically significant.

## Results

### Distribution of *TNRC9*, *FGFR2*, and *LSP1* SNPs in subjects

Using TaqMan SNP Genotyping Assays, we evaluated *FGFR2* rs2981582, *TNRC9* rs3803662, *TNRC9* rs12443621, and LP1 rs3817198 polymorphisms in 487 subjects, including 105 breast cancer cases and 382 controls. The genotype frequencies for these SNPs in cases and controls and the allele frequencies in all subjects are listed in Table 1. All four SNPs conformed to the Hardy-Weinberg equilibrium.

**Table 1:** Breast cancer risk in relation to selected SNPs

SNP	Genotypes	Cases	Controls	*χ* ^2a^	*P* ^a^	OR^a^	95 % CI^a^	Adjusted OR^b^	95 % CI^b^
TNRC9	AA	10 (11.2)	79 (18.8)	8.100	0.017	1.0		1.0	
rs12443621	AG	24 (22.86)	94 (24.61)			2.017	0.910–4.471	0.621	0.075–5.129
	GG	71 (67.62)	209 (54.71)			2.684	1.318–5.463	1.569	0.233–10.556
AG + GG		95 (90.48)	303 (79.32)						
G allele		0.79	0.670						
FGFR2	GG	50 (47.62)	120 (31.41)	10.203	0.006	1.0		1.0	
rs2981582	GA	27 (25.71)	146 (38.22)			0.444	0.262–0.752	0.557	0.141–2.199
	AA	28 (26.67)	116 (30.37)			0.579	0.342–0.983	0.559	0.154–2.032
GA + AA		55 (52.38)	262 (68.59)						
A allele		0.395	0.495						
TNRC9	CC	76 (72.38)	250 (65.44)	1.791	0.408	1.0		1.0	
rs3803662	CT	21 (20)	96 (25.13)			0.720	0.420–1.232	0.304	0.075–1.241
	TT	8 (7.61)	36 (9.42)			0.731	0.326–1.640	0.008	0.001–0.01
CT + TT		29 (27.62)	132 (34.56)						
T allele		0.176	0.219						
LSP1	TT	85 (80.95)	272 (71.20)	4.310	0.116	1.0		1.0	
rs3817198	TC	18 (17.14)	93 (24.35)			0.619	0.354–1.085	0.059	0.159–2.183
	CC	2 (1.90)	17 (4.45)			0.376	0.085–1.663	0.00	0.00–0.00
TC + CC		20 (19.05)	110 (28.80)						
C allele		0.104	0.166						

*SNPs* single nucleotide polymorphisms, *TNRC9* trinucleotide-repeat-containing 9, *FGFR2* fibroblast growth factor receptor 2, *LSP1* leukocyte-specific protein 1, *OR* odds ratio, *CI* confidence interval.

^a^Two-sided *χ* ^2^ test

^b^Age-adjusted

### Reproductive factors and breast cancer risk

Reproductive characteristics of cases and controls are summarized in Table 2. Case group had a significantly younger age at menarche compared to control group (*P* = 0.022). Controls had longer average breast-feeding duration (0.000) and higher rate of breast-feeding history (0.009). Controls also had a larger number of parity (0.03) and higher rate of childbearing history compared to cases (0.006). These factors were hence included in the multivariate analyses. All the other factors were similarly distributed between cases and controls.

**Table 2:** Comparison of age and reproductive factors between breast cancer cases and controls

	Cases (%)	Control (%)	*P* values	*χ* ^2^
Age	51.283 ± 11.29	49.91 ± 4.02	0.095	2.806
Age at menarche	14.53 ± 1.73	15.02 ± 1.99	0.022	5.291
Age at menopause	50.28 ± 4.14	49.07 ± 3.56	0.134	2.291
Breast-feeding (months)	9.03 ± 5.65	14.56 ± 11.71	0.000	34.31
No	25 (23.81)	50 (13.09)	0.009	6.895
Yes	80 (76.19)	332 (86.91)		
Childbearing			0.006	7.487
No	9 (8.57)	13 (3.40)		
Yes	96 (91.43)	369 (96.60)		
Parity			0.03	6.641
0	9 (8.57)	1 (0.26)		
1–2	93 (88.57)	345 (90.31)		
≥3	3 (2.86)	24 (6.28)		
Miscarriage/abortion			0.4	0.708
0	34 (32.38)	112 (29.31)		
≥1	71 (7.62)	270 (70.68)		
0	34 (32.38)	112 (29.32)	0.976	0.614
1–2	62 (59.05)	214 (56.02)		
≥3	9 (8.57)	56 (14.66)		

### *TNRC9*, *FGFR2*, and *LSP1* SNPs and breast cancer risk

We examined the association between *FGFR2* rs2981582, *TNRC9* rs3803662, *TNRC9* rs12443621, and LP1 rs3817198 and breast cancer risk, and the results are shown in Table 1. *FGFR2* rs2981582 and *TNRC9* rs12443621 genotype frequencies were significantly different between case and control groups (*P* = 0.006 and 0.017, respectively). The AG and GG genotypes of rs12443621 in *TNRC9* were more prevalent among cases than among controls (AG vs AA: OR = 2.017, 95 % CI = 0.910–4.471; GG vs AA: OR = 2.684, 95 % CI = 1.318–5.463). The GA and AA genotypes of rs2981582 in *FGFR2* were more prevalent among controls than among cases (GA vs GG: OR = 0.444, 95 % CI = 0.262–0.752; AA vs GG: OR = 0.579, 95 % CI = 0.342–0.983). After adjusting for age and reproductive risk factors, subjects carrying G allele of rs12443621 in *TNRC9* were found to have a higher breast cancer risk compared with those carrying AA genotype (AG vs AA: OR = 0.621, 95 % CI = 0.075–5.129; GG vs AA: OR = 1.569, 95 % CI = 0.233–10.556).

*TNRC9* rs3803662 and *LSP1* rs3817198 genotype distribution in cases and controls was not significantly different (*P* = 0.408 and 0.116, respectively).

### *TNRC9*, *FGFR2*, and *LSP1* SNPs and mammographic density

Analysis of mammographic density using rank sum test showed a significant difference between cases and controls (Table 3), with higher mammographic density in case patients (*χ*^2^ = 4.4530, *P* = 0.0348). As shown in Table 4, the distribution of *FGFR2* rs2981582 polymorphism, but not the other three SNPs, was significantly different among the four mammographic density categories (*P* = 0.0342). Subjects carrying AA genotype of rs2981582 in *FGFR2* were associated with reduced mammographic density (Table 5, *P* = 0.0092, 95 % CI = 0.334–0.926). After adjusting for age and reproductive risk factors, this remained significant (Table 6, *P* = 0.0154, 95 % CI = 0.294–0.893).

**Table 3:** Comparison of mammographic density between breast cancer cases and controls

	Category 1	Category 2	Category 3	Category 4	Total	*χ* ^*2*^	*P*
Cases	8(8.89)	27(30.00)	51(56.67)	4(4.44)	90		
Controls	8(3.49)	101(44.10)	114(49.78)	6(2.62)	229	4.453	0.035
Totals	16	128	165	10	319

**Table 4:** Mammographic density in relation to selected SNPs

SNP	Genotype	Mammographic density					*χ* ^*2*^	*P*
		Category 1	Category 2	Category 3	Category 4	Total		
TNRC9	AA	5(9.26)	27(50)	20(37.04)	2(3.70)	54	0.502	0.778
rs12443621	GG	7(3.83)	96(52.46)	74(40.44)	6(3.28)	183		
	AG	4(4.84)	42(51.22)	34(41.46)	2(2.44)	82		
	Total	16(5.02)	165(51.72)	128(40.13)	10(3.13)	319		
FGFR2	GG	8(6.72)	71(59.66)	36(30.25)	4(3.36)	119	6.752	0.0342
rs2981582	GA	4(3.64)	53(48.18)	49(44.55)	4(3.64)	110		
	AA	4(4.44)	41(45.56)	43(47.78)	2(2.22)	90		
	Total	16(5.02)	165(51.72)	128(40.13)	10(3.13)	319		
TNRC9	CC	14(6.45)	110(50.69)	85(39.17)	8(3.69)	217	1.1661	0.5582
rs3803662	CT	2(2.63)	38(50)	34(44.74)	2(2.63)	76		
	TT	0(0)	17(65.38)	9(34.62)	0(0)	26		
	Total	16(5.02)	165(51.72)	128(40.13)	10(3.13)	319		
LSP1	TT	10(4.17)	128(53.33)	92(38.33)	10(4.17)	240	0.654	0.7211
rs3817198	CT	4(5.80)	33(47.83)	32(46.38)	0(0)	69		
	CC	2(20)	4(40)	4(40)	0(0)	10		
	Total	16(5.02)	165(51.72)	128(40.13)	10(3.13)	319		

*SNPs* single nucleotide polymorphisms, *TNRC9* trinucleotide-repeat-containing 9, *FGFR2* fibroblast growth factor receptor 2, *LSP1* leukocyte-specific protein 1

**Table 5:** Logistic regression analysis of the relationship between mammographic density and selected SNPs

SNP	Genotype	*P*	OR	95 % CI
	AA		1.0	
TNRC9 rs12443621	AG	0.4876	0.802	0.444–1.452
	GG	0.7584	0.971	0.586–1.609
	GG		1.0	
FGFR2 rs2981582	GA	0.1929	1.022	0.596–1.751
	AA	0.0092	0.556	0.334–0.926
	CC		1.0	
TNRC9 rs3803662	CT	0.4619	0.826	0.372–1.834
	TT	0.3011	1.241	0.748–2.058
	TT		1.0	
LSP1 rs3817198	CT	0.3709	0.568	0.16–2.02
	CC	0.4326	1.031	0.614–1.733

*SNPs* single nucleotide polymorphisms, *TNRC9* trinucleotide-repeat-containing 9, *FGFR2* fibroblast growth factor receptor 2, *LSP1* leukocyte-specific protein 1, *OR* odds ratio, *CI* confidence interval

**Table 6:** Logistic regression analysis of the relationship between mammographic density and FGFR2 rs2981582 polymorphism

SNP	Genotype	*P*	OR^a^	95 % CI^a^
	GG		1.0	
FGFR2 rs2981582	GA	0.4296	0.884	0.489–1.597
	AA	0.0154	0.513	0.294–0.893

*SNPs* single nucleotide polymorphisms, *FGFR2* fibroblast growth factor receptor 2, *OR* odds ratio, *CI* confidence interval

^a^Adjusted for age, age at menarche, age at menopause, breast-feeding, parity, and miscarriage/abortions

## Discussion

In the present study, we found that early age at menarche is associated with increased breast cancer risk, whereas breast-feeding and parity are associated with reduced breast cancer risk. Multiple studies have examined the relationship between reproductive factors and breast cancer risk, but the results are somewhat inconsistent. A case-control study in Japanese population reported that early age at menarche, late age at first birth, and premenopausal status are significantly associated with breast cancer risk, while high parity is a protective factor [11]. A meta-analysis revealed that induced abortion is a risk factor for breast cancer [12]. Stuver and coworkers failed to observe any protective effects of lactation or duration of lactation against breast cancer risk [13], although there are other studies showing that lactation is linked to reduced risk of breast cancer [14]. Breast cancer is a complicated disease affected by genetic, environmental, and economic conditions as well as lifestyles. Variations in these factors may contribute to the inconsistency in research findings on the association between reproductive factors and breast cancer.

Our study revealed a significant association between *TNRC9* rs12443621 and breast cancer risk. Specifically, the GG genotype of *TNRC9* rs12443621 had increased risk of breast cancer. The rs12443621 SNP of *TNRC9* (also named TOX3) is located at chromosome 16q12 [4]. The function of *TNRC9* is unclear, but a recent paper has reported that *TNRC9* down-regulates BRCA1 expression and promotes breast cancer aggressiveness [7]. The association between *TNRC9* polymorphisms and breast cancer risk remains controversial. A meta-analysis in 2011 found that *TNRC9* rs3803662, but not *TNRC9* rs12443621, polymorphism was significantly correlated with breast cancer risk [15], which differs from our results. However, our findings agree with a recently published cohort study in Swedish population, which reports an increased risk of breast cancer for subjects carrying AG/GG genotypes of TRNC9 rs12443621 [6]. The reason for the inconsistency among published data regarding the association between TRNC9 rs12443621 and breast cancer risk is hard to decipher but could be related to the multifaceted characteristics of breast cancer.

*FGFR2* is a member of the FGFR family of receptor tyrosine kinases that has been found to be overexpressed in some breast cancer cell lines [16]. Amplification of *FGFR2* gene occurs in a small subset of breast cancer [17]. Our study reveals an interesting finding that the GA and AA genotypes of *FGFR2* rs2981582 are associated with reduced risk of breast cancer. This is surprising, since most studies reported that the T allele of *FGFR2* rs2981582 is associated with increased breast cancer risk [18, 19]. However, our results are consistent with another case-control study in the Chinese population, which also reveals the AA genotype of rs2981582 as a protective factor against breast cancer [20]. It is worthwhile to mention that we also observed a strong association between the GA and AA genotypes of *FGFR2* rs2981582 and decreased mammographic density. Meanwhile, our data showed that lower mammographic density is linked to lower risk of breast cancer. These findings are consistent with each other. One limitation of the current study was that the sample size for breast cancer cases was relatively small (105 cases were included in this study), and all patients were from Heilongjiang Province in Northeast China. We cannot rule out the potential instability rising from a relatively small number of patients. Further studies are necessary to elude the reasons for the disparity between our results and some of the earlier reports.

## Conclusions

In conclusion, we detected a significant association between the GG genotype of *TNRC9* rs12443621 and elevated breast cancer risk. Moreover, the GA and AA genotypes of *FGFR2* rs2981582 appear to be associated with lower mammographic density and reduced breast cancer risk. These data provide support for the potential importance of these polymorphisms in breast cancer biology. Further studies are needed to define the role of these SNPs in breast cancer and test the possibility of using them as biomarkers for diagnostic, prognostic, or therapeutic purposes in Chinese Han population.

## Abbreviations

CC: craniocaudal
CI: confidence intervals
*FGFR2*: fibroblast growth factor receptor 2
GWAS: genome-wide association studies
*LSP1*: leukocyte-specific protein 1
MAP3K1: mitogen-activated protein kinase 1
MLO: mediolateral oblique
OR: odds ratio
SNPs: single nucleotide polymorphisms
*TNRC9*: trinucleotide-repeat-containing 9
